# Improved performance and stability of perovskite solar cells with bilayer electron-transporting layers

**DOI:** 10.1039/c8ra00248g

**Published:** 2018-02-06

**Authors:** Tingting Jiang, Weifei Fu

**Affiliations:** The State Key Laboratory of Refractories and Metallurgy, College of Materials and Metallurgy, Wuhan University of Science and Technology Wuhan 430081 P. R. China; State Key Laboratory of Silicon Materials, MOE Key Laboratory of Macromolecular Synthesis and Functionalization, Department of Polymer Science and Engineering, Zhejiang University Hangzhou 310027 P. R. China zjufwf@zju.edu.cn

## Abstract

Zinc oxide nanoparticles (NPs) are very promising in replacing the phenyl-C_61_-butyric acid methyl ester (PC_61_BM) as electron-transporting materials due to the high carrier mobilities, superior stability, low cost and solution processability at low temperatures. The perovskite/ZnO NPs heterojunction has also demonstrated much better stability than perovskite/PC_61_BM, however it shows lower power conversion efficiency (PCE) compared to the state-of-art devices based on perovskite/PCBM heterojunction. Here, we demonstrated that the insufficient charge transfer from methylammonium lead iodide (MAPbI_3_) to ZnO NPs and significant interface trap-states lead to the poor performance and severe hysteresis of PSC with MAPbI_3_/ZnO NPs heterojunction. When PC_61_BM/ZnO NPs bilayer electron transporting layers (ETLs) were used with a device structure of ITO/poly(bis(4-phenyl)(2,4,6-trimethylphenyl)amine) (PTAA)/MAPbI_3_/PC_61_BM/ZnO NPs/Al, which can combine the advantages of efficient charge transfer from MAPbI_3_ to PC_61_BM and excellent blocking ability of ZnO NPs against oxygen, water and electrodes, highly efficient PSCs with PCE as high as 17.2% can be achieved with decent stability.

## Abstract 2

Perovskite solar cells with PC_61_BM/ZnO nanoparticles bilayer electron-transporting layers were achieved with a power conversion efficiency of 17.2% and decent stability.

## Graphical abstract

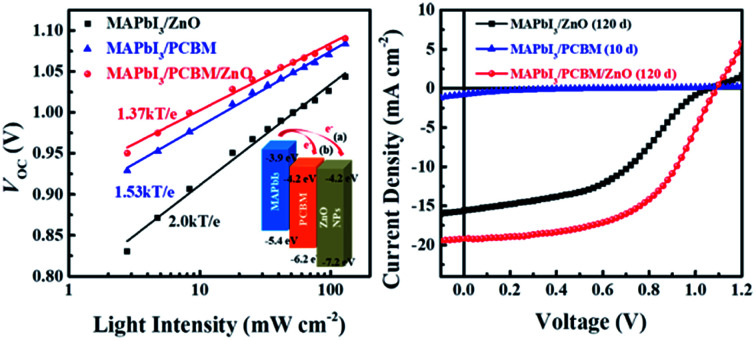

Organic–inorganic hybrid perovskite solar cells (PSCs) have recently attracted tremendous attention because of their excellent photovoltaic efficiencies.^1–4^ Since the initial results published in 2009 with efficiencies about 4% using a typical dye-sensitized solar cell structure with liquid electrolyte,^5^ significant progress has been made in device performance through developing high quality film processing methods,^6–10^ tuning the perovskite composition,^11–15^ optimizing the device architectures^16,17^ and synthesizing new hole/electron transport materials.^18–21^ Recently, a certified record power conversion efficiency (PCE) of 22.7% was achieved.^22^ Despite of the success in obtaining dramatically improved PCE, there are certain concerns about the stability and cost towards commercialization. For the state-of-the-art PSCs, perovskites are susceptible to degradation in moisture and air, thus the charge transport materials should prevent the perovskite from exposure to such environments.^20,23–25^ One the other hand, PSCs also suffer from the high cost of widely used organic charge transport materials such as 2,2,7,7-tetrakis(*N*,*N*-di-*p*-methoxyphenylamine)-9,9-spirobifluorene (spiro-OMeTAD), phenyl-C_61/71_-butyric acid methyl ester (PC_61/71_BM).^3,18,26^ As alternatives, inorganic materials such as CuSCN,^27^ CuI,^28^ CuGaO_2_,^20^ and NiO_*x*_ ^29,30^ which can be acted as hole transport materials and ZnO,^31,32^ SnO_2_ ^12,33,34^ and TiO_2_ ^10,35^ which can be acted as electron transport materials are widely studied. Among them, metal oxide nanoparticles (NPs) are very promising in replacing the organic counterparts due to the high carrier mobilities, superior stability, low cost and solution processability at low temperatures.^16,31,33^

The perovskite/ZnO NPs heterojunction has been demonstrated much better stability than perovskite/PCBM,^23^ however it shows lower PCE compared to the state-of-art devices based on perovskite/PCBM heterojunction.^36–38^ Thus in this paper, we systematically studied the charge transfer and recombination at CH_3_NH_3_PbI_3_ (MAPbI_3_) and ZnO NPs or PC_61_BM interfaces and tried to fabricate devices with high PCE and super stability simultaneously. We demonstrated that insufficient charge transfer from MAPbI_3_ to ZnO NPs and significant interface trap-states lead to the poor performance and severe hysteresis of PSCs based on MAPbI_3_/ZnO NPs heterojunction, while the devices based on MAPbI_3_/PC_61_BM show high PCE and negligible hysteresis due to the efficient charge transfer from MAPbI_3_ to PC_61_BM and less recombination at the interface. On the other hand, the MAPbI_3_/ZnO NPs devices show excellent stability in air because of the excellent capping ability of ZnO NPs while the stability of MAPbI_3_/PC_61_BM devices is very poor. Thus, we fabricated the PSCs with bilayer electron-transporting layers (ETLs) with the device structure of ITO/poly(bis(4-phenyl)(2,4,6-trimethylphenyl)amine) (PTAA)/MAPbI_3_/PC_61_BM/ZnO NPs/Al, trying to combine the advantages of efficient charge extraction ability of PC_61_BM and excellent blocking ability of ZnO NPs against oxygen, water and electrode, and finally device with PCE as high as 17.2% was achieved with decent stability.

## 1 Experimental

### 1.1 Materials

Zinc acetate dehydrate, tetramethylammonium hydroxide (TMAH), lead iodide, DMSO, DMF, chlorobenzene and toluene were purchased from Sigma-Aldrich and used as received. [6,6]-Phenyl-C_61_-butyric acid methyl ester (PC_61_BM) was purchased from American Dyes Source, Inc. CH_3_NH_3_I (MAI) was purchased from Shanghai Materwin New Materials Co. Ltd. Poly(bis(4-phenyl)(2,4,6-trimethylphenyl)amine) (PTAA) was purchased from Xi'an Polymer Light Technology Corporation. ZnO nanoparticles were synthesized by a sol–gel process using Zn acetate dehydrate and TMAH, and dispersed in anhydrous isopropanol with a concentration of 20 mg mL^−1^.^39^

### 1.2 Device fabrication and testing

Prior to fabrication, the substrates were cleaned by sonication using detergent, deionized water, acetone, and isopropanol sequentially for every 15 min followed by 15 min of ultraviolet ozone (UV-ozone) treatment. The substrates were transferred to a glovebox. PTAA film was fabricated by spin-coating a toluene solution with a concentration of 5 mg mL^−1^ on the ITO substrates in glove-box.

PbI_2_ (1 M) and DMSO (1 M) were dissolved in DMF under stirring at 70 °C. The solution was then spin coated on the PTAA film at 3000 rpm for 60 s. Then a solution of MAI in 2-propanol (IPA) (50 mg mL^−1^) was dropped and spin-coated at 3000 rpm for 60 s. Afterwards, the as prepared films were heated at 90 °C for 15 min. After cooling down, a layer of PC_61_BM (20 mg mL^−1^ in chlorobenzene) was spin-coated at 2000 rpm for 45 s for MAPbI_3_/PCBM junction solar cells. While for MAPbI_3_/ZnO junction solar cells ZnO nanoparticles in isopropanol was spin-coated at 4000 rpm for 30 s. Subsequently, samples were loaded into a vacuum deposition chamber (background pressure ≈ 5 × 10^−4^ Pa) to deposit a 100 nm thick Al cathode with a shadow mask. To specify the illuminated area, we used an aperture with an area of 0.06 cm^2^, whereas the total device area defined by the overlap of the electrodes was approximately 0.12 cm^2^.

The *J*–*V* characteristics were measured with Keithley 2400 measurement source units with the devices maintained at room temperature in glove-box. The photovoltaic response was measured under a calibrated solar simulator (Enli Technology) at 100 mW cm^−2^, and the light intensity was calibrated with a standard photovoltaic reference cell. The devices were stored in glove-box in dark overnight before measurement. The forward *J*–*V* scans were measured from −0.1 V to 1.2 V with a scan rate of 0.05 V s^−1^ and a voltage step of 0.01 V while the reverse *J*–*V* scans were measured from 1.2 V to −0.1 V with a scan rate of 0.05 V s^−1^ and a voltage step of 0.01 V. The EQE spectrum was measured using a QE-R Model of Enli Technology.

## 2 Results and discussion

**Fig. 1:**
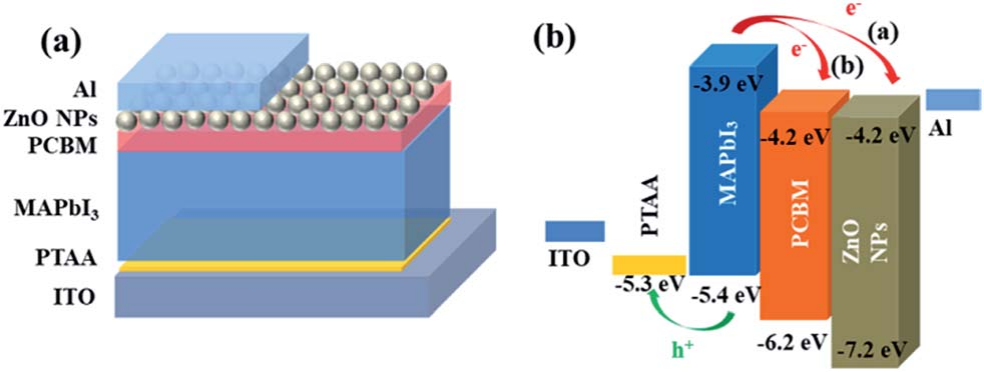
Schematic illustration of the perovskite solar cell configuration with PC_61_BM/ZnO NPs as ETL (a). (b) The corresponding energy level diagram of the devices.

**Fig. 2:**
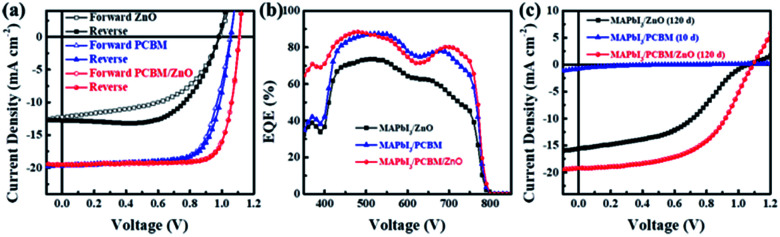
Current density–voltage (*J*–*V*) curves (a) and external quantum efficiencies (b) of fresh perovskite solar cells with different ETLs. (c) *J*–*V* curves of the devices after storage with a period of 120 days in air with a humidity of 20%. MAPbI_3_/PC_61_BM heterojunction solar cell was only stored 10 days in the same condition.

Inverted perovskite solar cells with the device architecture of ITO/PTAA/MAPbI_3_/PC_61_BM or ZnO NPs/Al were fabricated, which were shown in Fig. 1a. Fig. 1b shows the corresponding energy level diagram of the devices. The reported valance band of ZnO NPs and PC_61_BM are similar with a value of 4.2 eV, which is 0.3 eV lower the valance band of MAPbI_3_, and thus the electrons in perovskite film can transfer to both ETLs and be collected by electrodes. Fig. 2a shows the current–voltage (*J*–*V*) characteristics of PSCs based on ZnO NPs and PC_61_BM as ETLs under 100 mW cm^−2^ AM1.5G solar illumination with reverse and forward scans. The corresponding photovoltaic parameters are summarized in Table 1. The device employing ZnO as ETL exhibits an open-circuit voltage (*V*_OC_) of 0.98 V, a short-circuit current density (*J*_SC_) of 12.5 mA cm^−2^, and a fill factor (FF) of 0.64, yielding a PCE of 8.0% at reverse scan, and suffers severe hysteresis with a much lower PCE of 6.3% (*V*_OC_ = 0.99 V, *J*_SC_ = 12.0 mA cm^−2^ and FF = 0.52) at forward scan, respectively. While the device using PC_61_BM as ETL shows a significant improvement in PCE up to 15.0% at reverse scan with a *V*_OC_ of 1.06 V, a *J*_SC_ of 19.1 mA cm^−2^ and a FF of 0.72, and more importantly, with negligible hysteresis (14.70% PCE at forward scan with a *V*_OC_ of 1.05 V, a *J*_SC_ of 19.2 mA cm^−2^ and a FF of 0.71). It exhibits improvement on all three parameters simultaneously compared to those of ZnO NPs based devices. The much enhanced *J*_SC_ was also demonstrated by the external quantum efficiency (EQE) spectra shown in Fig. 2b.

**Table 1:** Device parameters of the PSCs with different ETLs under the illumination of AM 1.5G, 100 mW cm^−2^

ETL	Scan direction	*V*_OC_ [V]	*J*_SC_ [mA cm^−2^]	FF	PCE [%]
ZnO NPs	Forward	0.99	12.0	0.52	6.3
	Reverse	0.98	12.5	0.64	8.0
PCBM	Forward	1.05	19.2	0.71	14.7
	Reverse	1.06	19.1	0.72	15.0
PCBM/ZnO	Forward	1.11	19.5	0.78	16.9
	Reverse	1.11	19.6	0.79	17.2

We also tested the stability of devices which were stored in air under dark with a humidity of 20% for 120 days. The corresponding *J*–*V* curves were shown in Fig. 2c. We found that the device with ZnO NPs as ETL retained 95% of the initial PCE, with a *V*_OC_ of 1.06 V, a *J*_SC_ of 15.6 mA cm^−2^, a FF of 0.45 and a PCE of 7.6% after 120 days storage, while the device with only PC_61_BM as ETL almost died only after 10 days, showing a *V*_OC_ of 0.56 V, a *J*_SC_ of 0.77 mA cm^−2^, a FF of 0.13 and a PCE of 0.06%. The much worse stability was attributed to the poor blocking ability of PC_61_BM against oxygen, water and the electrode.^23^

In order to obtain both high PCE and excellent stability, we also fabricated device with bilayer ETLs in a structure of ITO/PTAA/MAPbI_3_/PC_61_BM/ZnO NPs/Al shown in Fig. 1a. The device shows highest *V*_OC_, *J*_SC_, FF and PCE which ups to 17.2% at reverse scan with a *V*_OC_ of 1.11 V, a *J*_SC_ of 19.6 mA cm^−2^ and a FF of 0.79, and also shows negligible hysteresis with a *V*_OC_ of 1.11 V, a *J*_SC_ of 19.5 mA cm^−2^, a FF of 0.78 and a PCE of 16.9% at forward scan. After stored in air 120 days, the device also shows a decent PCE of 11.4%, with a *V*_OC_ of 1.09 V, a *J*_SC_ of 19.2 mA cm^−2^, a FF of 0.54, which is 66% of the initial value. Thus we demonstrated that this type of device show the advantages of the MAPbI_3_/PC_61_BM device with high PCE and the MAPbI_3_/ZnO NPs device with good stability.

**Fig. 3:**
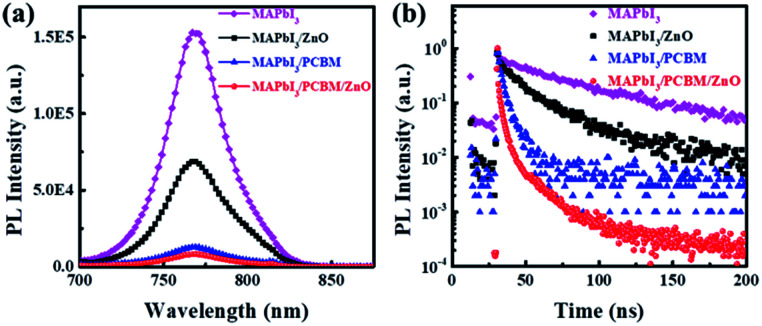
(a) The steady-state photoluminescence (PL) spectra of MAPbI_3_, MAPbI_3_/ZnO NPs, MAPbI_3_/PC_61_BM and MAPbI_3_/PC_61_BM/ZnO films. The excitation light was set at 610 nm. (b) Time-resolved PL measurements taken at the peak emission wavelength (765 nm) of the various perovskite films. A 5 mW picosecond pulsed diode laser at 638.8 nm excited on glass side of films.

The effects of different ETLs on the charge extraction and recombination process at perovskite/ETL interface were investigated in details to find out the reasons of different behaviours of corresponding devices. Steady-state photoluminescence (PL) was performed to compare the electron transfer efficiency from perovskite to ETLs. As shown in Fig. 3a, only 54% of the PL intensity was quenched by depositing ZnO NPs on top, while more than 90% PL intensity was quenched when PC_61_BM or PC_61_BM/ZnO NPs were deposited on top of the perovskite layer. This means insufficient charge transfer from perovskite to ZnO NPs, while electrons in perovskite film can be efficiently transferred to PC_61_BM layer. This was further confirmed by time-resolved PL (TRPL) (Fig. 3b). The TRPL curve was fitted to a biexponential equation: *Y* = *A*_1_ exp(−*t*/*τ*_1_) + *A*_2_ exp(−t/*τ*_2_) and the detailed data are shown in Table 2. In the absence of ETL quencher, the pristine perovskite film showed a relatively long PL lifetime of 56.8 ns, while it decreased to 25.6, 5.4 and 5.3 ns for the ZnO NPs, PC_61_BM and PC_61_BM/ZnO NPs-based films, respectively. This implies that faster and more efficient electron extraction was achieved at the perovskite/PC_61_BM interface. The insufficient charge transfer from perovskite to the ZnO NPs layer compared to PC_61_BM could due to the shallower conduction band and lower electron mobility of ZnO NPs, or the interfacial traps at the perovskite/ZnO NPs interface,^23^ and the worse contact at perovskite/ZnO NPs interface. These could cause charge accumulation at the perovskite/ZnO NPs interface and thus leads to poor performance and severe hysteresis in the corresponding device.^23,33,40^

**Table 2:** Time-resolved PL data of perovskite films on glass with various ETLs

Sample	*τ*_1_ [ns]	Frac. [%]	*τ*_2_ [ns]	Frac. [%]	Average [ns]
MAPbI_3_	10.0	4.2	58.9	95.8	56.8
MAPbI_3_/ZnO	9.3	34.0	34.0	66.0	25.6
MAPbI_3_/PCBM	2.3	61.5	10.4	38.5	5.4
MAPbI_3_/PCBM/ZnO	1.6	68.5	13.4	31.5	5.3

**Fig. 4:**
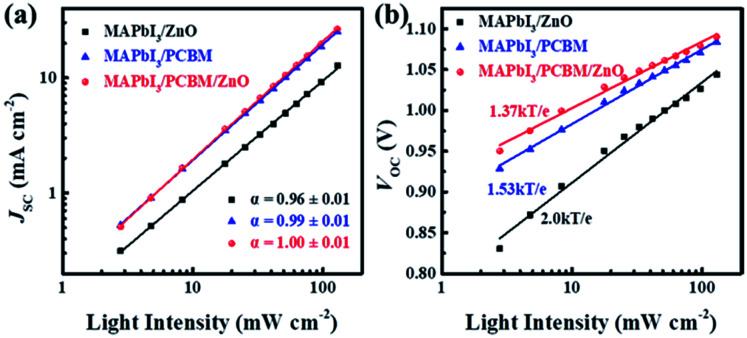
(a) Measured *J*_SC_ of cells with different ETLs plotted against light intensity (symbols) on a logarithmic scale. Fitting a power law (solid lines) to these data yields *α*. (b) Measured *V*_OC_ of cells with different ETLs plotted against light intensity (symbols), together with linear fits to the data (solid lines).

The recombination kinetics were also studied carefully by measuring *J*_SC_ and *V*_OC_ at various light intensities (*I*) from 130 to 2.8 mW cm^−2^ (Fig. 4a and b). A power law dependence of *J*_SC_ upon illumination intensity is generally expressed as *J*_SC_ ∝ *I*^*α*^, where *I* is the light intensity and *α* is the exponential factor. At short circuit condition, the bimolecular recombination should be minimum (*α* ≈ 1) for maximum carrier sweep out. Any deviation from *α* ≈ 1 implies bimolecular recombination.^41–43^Fig. 4a shows that *J*_SC_ ∝ *I*^*α*^, where *α* = 0.96 ± 0.01 for the device using ZnO NPs as ETL while *α* ≈ 1 for both devices using PC_61_BM or PC_61_BM/ZnO NPs as ETLs, indicating weak bimolecular recombination at short-circuit condition in the latter two types of devices.^43^ For the MAPbI_3_/ZnO NPs device, the lower *α* could be attributed to the bimolecular recombination during sweep-out.^41,42^ At open-circuit conditions, the current is zero, all carriers recombine within the cell. Thus, recombination studies near open circuit are particularly sensitive to the details of the recombination mechanism.^43^Fig. 4b shows *V*_OC_ varies logarithmically (ln(*I*)) with light intensity. The slopes for device using ZnO NPs, PC_61_BM and PC_61_BM/ZnO NPs as ETLs are 2.0*kT*/*e*, 1.53*kT*/*e* and 1.37*kT*/*e* respectively, where *k* is the Boltzmann constant, *T* is absolute temperature, and *e* is elementary charge. In principle, the slope of *V*_OC_*versus* light intensity will be equal to *kT*/*e* if device without trapping of charge carriers or governed by bimolecular recombination, which refers to the recombination of free electrons and holes in the photoactive layer.^20,41,43^ The slope of ZnO NPs based device is 2.0*kT*/*e*, implying that the monomolecular recombination (Shockley–Read–Hall) recombination through trap states or recombination centers is dominant even at open circuit, which leading to the reduced *V*_OC_ and severe hysteresis.^36,44^ In the MAPbI_3_/PC_61_BM and MAPbI_3_/PC_61_BM/ZnO NPs devices, the slopes indicate that recombination at open circuit is a combination of monomolecular and bimolecular process in both cases and the smaller slopes imply reduced SRH recombination, which contribute to the negligible hysteresis and high device performance. The smallest slope with PC_61_BM/ZnO NPs bilayer ETLs was also attributed the better contact at ZnO/Al interface except for MAPbI_3_/PCBM interface because the robust ZnO NPs film can prevent the Al diffusing into perovskites.^23^ The reduced recombination at both interfaces renders the highest *V*_OC_ and performance of this type device.

## 3 Conclusions

We demonstrated that the insufficient charge transfer from MAPbI_3_ to ZnO NPs and significant interface trap-states lead to the poor performance and severe hysteresis of PSC with MAPbI_3_/ZnO NPs heterojunction, but the device shows super stability in air. While the device based on MAPbI_3_/PC_61_BM heterojunction shows high PCE and negligible hysteresis due to the efficient charge transfer from MAPbI_3_ to PC_61_BM and less recombination at the interface, however the device show very poor stability in air. When PC_61_BM/ZnO NPs bilayer ETLs were used with a device structure of ITO/PTAA/MAPbI_3_/PC_61_BM/ZnO NPs/Al, high efficient PSCs with PCE as high as 17.2% can be achieved with decent stability. Our study also showed the possibility of obtaining highly efficient perovskite/metal oxide NPs heterojunction solar cells by interface engineering without high cost PC_61_BM.

## Conflicts of interest

There are no conflicts to declare.

## Supplementary Material
